# Timing matters: traffic noise accelerates telomere loss rate differently across developmental stages

**DOI:** 10.1186/s12983-018-0275-8

**Published:** 2018-08-28

**Authors:** A. M. Dorado-Correa, S.A. Zollinger, B. Heidinger, H. Brumm

**Affiliations:** 1 Max Planck Institute for Ornithology, Eberhard-Gwinner-Str. 11, 82319 Seewiesen, Germany; 2 North Dakota State University, Stevens Hall 225, Dept 2715, PO Box 6050, Fargo, ND 58108-6050 USA

**Keywords:** Ageing, Early-life adversity, Early-life stress, Ecological novelty, Global change, Telomeres, Traffic noise, Urbanization, Zebra finches

## Abstract

**Background:**

Noise pollution is one of the leading environmental health risks for humans, linked to a myriad of stress-related health problems. Yet little is known about the long-term effects of noise on the health and fitness of wildlife. We experimentally investigated the direct and cross-generational effects of traffic noise on telomeres; a measure of cellular ageing that is predictive of disease and longevity in humans and other organisms. We exposed zebra finches (*Taenopygia guttata*) to three different treatment groups: 1) parents were exposed to traffic noise before and during breeding, together with their nestling young, 2) fledged juveniles but not their parents were exposed to traffic noise, and 3) control group birds were never exposed to traffic noise.

**Results:**

Although there was no significant effect of traffic noise exposure at early (pre-fledging) stages of offspring telomere length or loss rate, traffic noise exposure accelerated telomere loss in older (post-fledging) juveniles.

**Conclusions:**

The age-dependent differences found in this study in telomere loss could occur if parents buffer younger offspring against the detrimental effects of noise exposure and/or if younger offspring are less sensitive to noise exposure. Telomere length during early life has been shown to be positively related to lifespan and the observed noise-induced increase of telomere attrition rate could reduce the fitness of the affected birds and potentially alter the population dynamics of birds in noise polluted areas. Our data highlight the need to consider the developmental stage of an organism to better understand the ecological consequences of anthropogenic change.

**Electronic supplementary material:**

The online version of this article (10.1186/s12983-018-0275-8) contains supplementary material, which is available to authorized users.

## Background

Rapid environmental change due to urbanization, can be detrimental for many organisms, including humans [1–3]. The growth of urban areas is linked to severe environmental contamination, including chemical, light, and noise pollution and these anthropogenic changes can often be regarded as environmental stressors [4]. For example, in humans and other mammals, noise is related to delays in brain development, impaired cognitive function and deficits in learning and memory as well as high blood pressure, hyperglycemia, and elevated cholesterol levels [5–12]. However, to understand the causal effects of urbanization on organisms it is important to identify model systems that can be experimentally manipulated. Such investigations are necessary to disentangle the impacts of different environmental factors associated with urban habitats on the development, health, behaviour, and fitness of exposed individuals.

Moreover, the mechanisms that underlie the negative effects of increased noise exposure on health and fitness are poorly understood. In diverse organisms, environmental conditions experienced during early life often have delayed impacts on phenotypic development and fitness [13, 14]. Telomere loss is a mechanism that may provide a link between early stress exposure and longevity [15]. Telomeres are non-coding, repetitive DNA sequences that cap the ends of eukaryote chromosomes and enhance genome stability [16]. Telomeres shorten during cell division and limit cellular lifespan [16] and telomere length has often been shown to be positively associated with longevity [17]. Telomeres have also been shown to shorten in response to stress in mammals and some birds [17–19].

In humans and in birds, there is also evidence that stress experienced by parents can impact offspring telomere length and loss rate [20–29]. For example, human mothers that reported experiencing stressful conditions during pregnancy produced offspring with shorter telomeres at birth [22] and in adulthood [21]. As these studies are necessarily correlative it is difficult to separate cause and effect. But, these findings are also supported by a recent experimental study in birds where experimentally elevated stress hormone levels in the yolk resulted in chicks with shorter telomeres at the end of post-natal development [20]. However, the relative importance of stress exposure experienced by parents and offspring at different developmental stages has rarely been disentangled.

Information regarding effects of urbanization on telomere length or attrition rates in birds is still scarce. However, two recent studies have found links between urbanization and telomere length in juvenile songbirds. In a cross-fostering experiment, it was found that great tits (*Parus major*) reared from 2 days of age in urban environments had significantly shorter telomeres at 15 days of age than birds reared in rural areas [30]. It remains unclear which aspects of urbanization may have contributed to this effect of living in an urban environment on telomere dynamics. In another study, playback of traffic noise in the field resulted in shorter telomeres in 9-day-old house sparrows (*Passer domesticus*) [31]. In addition, one field study showed that telomere length may predict post-fledging survival and recruitment of great tits in urban and rural areas. However, in urban environments adult great tits had longer telomeres, on average than tits in rural populations, possibly due to selective disappearance of individuals with shorter telomeres in early life [32]. Yet, neither of these studies investigated whether exposure to traffic noise had longer-term effects on telomere attrition rates, or whether chronic noise exposure affects individuals at different ontogenetic stages.

We experimentally tested for direct and cross-generational effects of traffic noise exposure across developmental stages in zebra finches bred in aviaries in our laboratory. In this experiment we compared telomere lengths at 21 and 120 days post-hatch in (1) birds that hatched to parents that were exposed to noise during courtship, egg-laying, and nestling care periods, with the offspring themselves also exposed to noise until ca. 18 days post-hatch, (2) birds that hatched to non-noise exposed parents, but which were themselves exposed to noise from day 18 to 120, and (3) controls in which neither the parents nor the chicks were exposed to noise. If physiological stress responses in female birds during courtship and egg-laying are elevated due to chronic noise exposure, their eggs could contain more maternal glucocorticoids than those of non-stressed females. Therefore, we hypothesized that the offspring of noise-stressed females may have elevated levels of glucorcorticoids and/or oxidative damage, both of which could result in shorter telomeres. If the effects of noise as a stressor are direct, rather than cross-generational, we predicted that offspring who were chronically exposed to noise themselves would suffer higher rates of telomere loss, but those whose parents had noise exposure would not suffer the same degree of telomere damage.

## Methods

### Study system

We bred adult zebra finches (2–3 years old) from the colonies at the Max Planck Institute for Ornithology in Seewiesen, Germany. Each of three experimental rooms consisted of three aviaries (1 × 2 × 2 m), each housing 7–8 pairs of birds. Each aviary was provided with 12 wooden nest boxes and ad libitum nesting materials, seeds, commercial finch egg food and water. In addition, birds were provided with fresh vegetables and hard-boiled eggs twice weekly throughout the experimental period. Our experimental birds were the offspring of these breeding adults. Each aviary produced an average of 29 offspring (16–42), with a total of 263 offspring from all treatment groups. Offspring that died before they reached 120 days post-hatch were not included in the experiment. Animal housing and care was all in accordance with European and local laws governing the care and use of laboratory animals (Council of Europe Treaty ETS-123). All experimental procedures were approved by and done under license from the Government of Upper Bavaria (Regierung von Oberbayern), licence number 55.2–1–54-2532-51-2013.

### Experimental treatment

To determine if typical city traffic noise affects telomere dynamics in juvenile birds, we designed three noise exposure treatments: 1) the parents were exposed to noise during breeding, egg-laying and nestling care periods (PNoise), 2) juvenile birds were exposed to noise exposure from fledging throughout the sensory motor learning period, 18–120 days post-hatch (JNoise), and 3) a control group that was not exposed to noise at any time point (NoNoise) (Additional file 1: Table S1). Thus, the offspring in the PNoise treatment group were not exposed to traffic noise after fledging, and the offspring in the JNoise treatment were not exposed before fledging, nor were their parents. The PNoise group had a total of 95 offspring from 32 broods, the JNoise group 59 from 17 broods, and the control group (NoNoise) had 109 offspring from 35 broods. The difference in sample size between treatments is because the parents of the treatments PNoise and NoNoise bred twice, once in PNoise and once in NoNoise treatments. To control for potential effects of breeding experience we considered the number of breeding rounds in the statistical analysis (see below).

Noise playback consisted of 80, 5-min long recordings of street traffic noise, which was recorded at several busy intersections in Munich, Germany during April 2013. During the daylight hours (06:30–20:30), the 80 recordings were played continuously, in randomized order, with playback levels (measured at the position of the nest boxes) averaging between 65 and 85 dB(A) re 20 μPa. Nighttime playback (20:30–06:30) consisted of randomized playback of 40 noise recordings, which were less dense in the rate of passing than the daytime recordings and were reduced in overall amplitude, with playback level averages ranging between 45 and 75 dB(A). Therefore, noise playback mimicked typical urban noise patterns, according to published noise maps [33]. We played noise from a laptop computer to an array of 12 pairs of amplified portable speakers (Hama Sonic Mobil 400 Alu PS1032), with 4 pairs arranged above each of the three aviaries in the room. Noise playback was run using a script written in MatLab (version 7.5.0; Natick, MA, USA; www.mathworks.com) to randomize playback during day and night. For the PNoise group, playback of noise began 4 weeks before the introduction of nesting materials and nest boxes and continued until the median juvenile in the room had fledged (the date when half of the offspring had fledged). For the JNoise group, the noise playback began when the median juvenile was 18 days post-hatch, and continued until all juveniles had reached 120 days.

### Telomere measurement

Blood samples were collected by brachial venipuncture for each bird at 21 and 120 days post-hatch to measure telomere length and loss rate. Telomere length at 25 days has previously been shown to be positively related to lifespan in zebra finches [17]. Blood was collected into heparinized capillary tubes (1.4 × 75 mm), transferred into Eppendorf tubes, and centrifuged to separate the cells from the plasma. The cells were then stored at − 80 °C until DNA extraction. We analyzed samples for 263 birds in total, 137 females, and 126 males, at both ages. We used the DNeasy Blood and Tissue kit (Qiagen) to extract genomic DNA from the red blood cells following the manufacturer’s protocol. We used a NanoDrop 8000 spectrophotometer (Thermo Scientific) to measure the quantity of the DNA. To measure relative telomere length we used quantitative PCR (Stratagene MX3000P), as described in [34], and adapted to zebra finches [35].

The relative telomere length of each sample was measured by calculating the ratio (T/S) of telomere repeat copy number (T) to single control gene copy number (S), relative to a reference sample. As the control gene, we used the Glyceraldehyde-3-phosphate dehydrogenase (GAPDH). The following forward and reverse primers were used to amplify the telomere: Tel1b (5′-CGGTTTGTTTGGGTTTGGGTTTGGGTTTGGGTTTGGGTT-3′), Tel2b (5′-GGCTTGCCTTACCCTTACCCTTACCCTTACCCTTACCCT-3′) and zebra finch-specific GAPDH sequences: GAPDH-F (5′-AACCAGCCAAGTACGATGACAT-3′), GAPDH-R (5′-CCATCAGCAGCAGCCTTCA-3′). The telomere and GAPDH reactions were carried out on different plates, the number of PCR cycles required for the products to accumulate enough fluorescent signals to cross a threshold was determined. The detailed description of the conditions of the PCR can be found in [35]. A standard curve was included to measure the efficiencies of the reactions on every plate. The reference sample was from a zebra finch that was 21 days old at the time of collection. The efficiencies were within an acceptable range (plate mean ± SD GADPH 100.82 ± 3.31; telomere 91.68 ± 6.47) in all cases. All samples, including the standard curve, were run in triplicate, and average values were used to calculate the relative T/S ratios for each sample relative to the reference sample (for details see [17]). All of the samples of an individual were run on the same plate, i.e. the samples from each individual, taken on day 21 and 120 were run in the same plate in triplicate. In total, 23 plates were run for telomeres and GAPDH. The mean ± SD intraplate coefficient of variation of the Ct values was calculated per plate by dividing the standard deviation by the mean of the 20 ng concentration wells in the standard curve, multiplied by 100 (3 replicates). As a result, we got 1.99 ± 1.00 intraplate variation for the telomere assays and 0.15 ± 0.09 for the GAPDH assays, respectively. The average interplate variation for the ΔCt values was 3.96% and was calculated using the standard deviation value of ΔCt of the 20 ng wells of the standard curve of all plates divided by the mean, multiplied by 100.

### Paternity analysis

To account for possible genetic effects on telomere loss, we considered the identity of parents in the analysis (see below). Since there is typically a considerable amount of extra pair young in captive zebra finch colonies [36], genetic paternity analysis is necessary to reliably assign parentage. To this end, all offspring were genotyped at 11 highly polymorphic microsatellite markers [37] and parentage was assigned by exclusion.

### Statistics

All statistical analyses were performed with R 3.1.1 (R Core Team 2013). We fitted linear mixed-effects models to analyze our data, using the “lmer” function (package lme4). Additionally, we used the “sim” function (package arm) to simulate the posterior distribution of the model parameters and values were extracted based on 2000 simulations [38]. The statistical significance of fixed effects and interactions were assessed based on the 95% credible intervals (CI) around the mean (estimate). We considered an effect to be “significant” in the frequentist’s sense (*p* < 0.05) when the 95% CI did not overlap zero [39]. Telomere length (log-transformed) was set as the dependent variable, treatment (NoNoise, PNoise, JNoise), age when the sample was taken (21 or 120 days old), sex, mass of every individual at 21 and 120 days (mass) and breeding round as independent factors. Breeding round is the number of times the adults have reproduced. The individual ID, the ID of the genetic parents, and the aviary (to account for effects of the common aviary) were included as random effects. Genetic parentage was determined by exclusion using the R package SOLOMON [40]. The model used in the paper included interaction between treatment and age and was compared to other models using the Akaike Information Criterion (AIC), REML was set to FALSE [41]. The repeatability was calculated based on random effects of the model, for the individual repeatability parents ID were not taken into account.

## Results

**Fig. 1:**
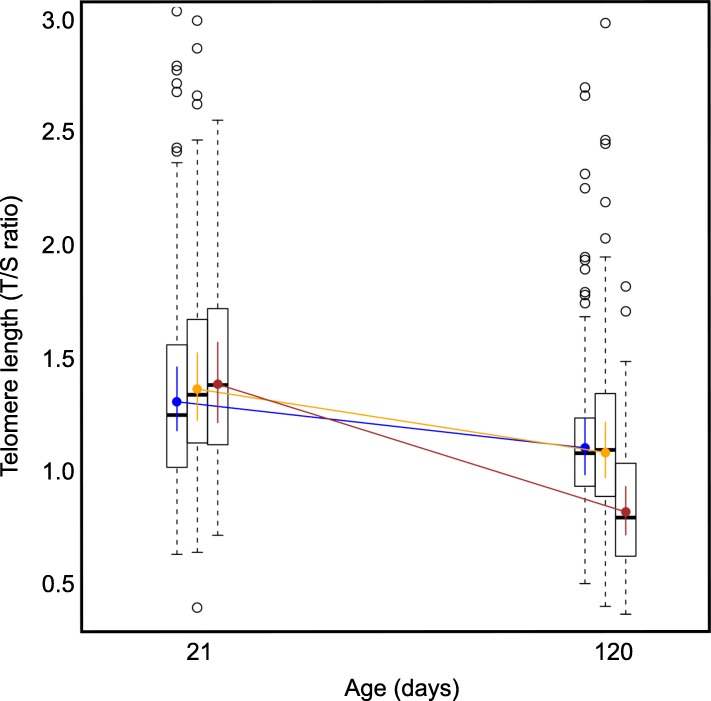
Posterior mean estimates of telomere length values of zebra finches with parents exposed to noise (orange circles), juveniles exposed to noise themselves (red circles) and control (blue circles), error bars around these coloured points indicate 95% confidence intervals. Box plots behind the coloured points represent the data points of the telomere length in the different treatments. The horizontal line is the median, the box contains 50% of the data points, the whiskers give the range of the data points within a distance of 1.5 times the interquartile range from the edge of the box and the white circles are outliers

Telomere length decreased with age in all treatment groups (Table 1, Fig. 1). At 21 days, telomere lengths did not significantly differ between treatments (mean ± SD NoNoise: 1.40 ± 0.55 T/S ratio; PNoise: 1.46 ± 0.53 T/S ratio; JNoise: 1.46 ± 0.50 T/S ratio). The JNoise group experienced significantly greater telomere shortening between days 21 and 120 days than the PNoise and NoNoise groups (Table 1, Fig. 1 and Additional file 1: Figure S1). Zebra finch juveniles exposed to noise post-fledging (JNoise treatment) had shorter telomeres at 120 days (mean ± SD: 0.87 ± 0.33 T/S ratio) than the offspring from the PNoise treatment (mean ± SD: 1.17 ± 0.42 T/S ratio) and NoNoise treatment (mean ± SD: 1.19 ± 0.53 T/S ratio) groups. The estimate of individual repeatability was 0.38 CI 0.27, 0.47. The estimate of mother repeatability was 0.19 CI 0.07, 0.31 and father repeatability was 0.08 CI 0.05, 0.29. The values of telomere length at 21 days in all three groups were within the range of telomere lengths found in previous studies in zebra finches [17]. There were no significant effects of sex, breeding round, or body mass on telomere length (Table 1).

**Table 1:** Outcome of linear models testing the effects of noise on the telomere length of juvenile zebra finches that had parents exposed to noise (PNoise), or that were themselves exposed to noise (JNoise) and a no-noise control group. The asterisks represent “significant” differences in the frequentist’s sense, i.e. when the 95% credible intervals did not overlap zero [39]

Parameters	Estimate (β)	95% CI
Fixed effects		
(Intercept)	0.11	−0.16,0.37
Parents in noise (PNoise)	0.06	−0.01,0.12
Offspring in noise (JNoise)	0.04	−0.07,0.15
Sex	0.03	−0.02,0.08
Age	−0.2	−0.27,-0.12*
Breeding round	0.03	−0.03,0.08
Mass	0.01	−0.007,0.03
PNoise x age	−0.06	− 0.15,0.02
JNoise x age	−0.32	− 0.60,-0.02*
Random effects	Std. Dev (σ^2^)	
Individual ID (Intercept)	0.04	
Mother ID (Intercept)	0.08	
Father ID (Intercept)	0.09	
Group (Intercept)	0.01	
Plate	0.23	
Residual	0.22

## Discussion

This study is the first to experimentally examine the effects of traffic noise on telomere length and attrition rate under controlled laboratory conditions. We found that traffic noise had a direct impact on the rate of telomere loss in post-fledge juvenile zebra finches. However, there were no significant effects of noise experienced by parents, or directly by nestlings at an earlier developmental stage (pre-fledging). At day 21 post-hatch, birds from the three different treatments did not differ significantly in telomere length. Importantly though, we found that the juveniles directly exposed to noise during the post-fledging period (18 to 120 days post-hatch) experienced significantly more telomere shortening and had significantly shorter telomeres at 120 days than birds in the control treatment, or those whose parents were exposed to noise. Therefore, noise exposure during the later stages of ontogeny (between 18 and 120 days) resulted in an increased rate of telomere loss. We did not find significant differences in telomere length or attrition rates between sexes. Our data suggest that juveniles that experience traffic noise will have greater telomere attrition, which may serve as a biomarker that predicts reduced longevity with negative consequences for fitness.

The pattern of noise-induced telomere loss in older juveniles may suggest that zebra finches are less sensitive to traffic noise when exposure occurs while they are still in the nest. One explanation is that the expression of telomerase, the enzyme that repairs telomeres, may be increased during some developmental stages. In zebra finches, telomerase activity is highest during the hatchling age period, when the proliferative demands of most organs are the highest [42], which may mean that very young birds are buffered against stress-induced telomere loss by higher rates of repair, compared to older juveniles. However, two field studies on other songbird species have found differences in telomere length in response to noise exposure or urbanization as early as day 9 and day 15 [30, 31]. Alternatively, it could be that the effect was more pronounced during young adulthood in zebra finches because the period between18 and 120 days post-hatch is a critical period. Zebra finches typically leave the nest around day 18 and continue to be fed entirely or partially by their parents or other adults until approximately 30 days post-hatch when juveniles become independent from their parents. In addition to the stress of weaning from parental nutritional support, this time period (ca. day 20) is approximately when zebra finches enter the song learning period [43]. This could mean that birds are more sensitive to noise or that it is a more potent stressor during this stage, than at earlier life stages. Corroborative evidence for this notion comes from a recent study that found that noise exposure affects the size of brain regions associated with song learning in zebra finches during their song learning period although it did not affect corticosterone levels [44].

There are many reasons that noise experienced by parents or by offspring at earlier stages may not have affected offspring telomeres. For example if parents in noisy environments make behavioural changes, this could potentially mitigate any negative effects of noise exposure on telomere loss to their offspring and/or if parents habituate to noise in the weeks before eggs are produced. Previous studies have found that parental behaviours often change in the presence of noise [45] which can reduce reproductive success [46, 47] . In our study, it could be that parents exposed to traffic noise found ways to alleviate the effect of noise on their offspring. Such mitigation could have been mediated by increased parental care behaviour or even earlier in the gamete formation stage. For example, if young chicks (< 18 days) experienced higher rates of telomere loss than controls, noise-exposed parents may have produced offspring with longer telomeres at hatching, which were then lost at a higher rate during their first 20 days post-hatch. Thus, this rapid change may be indistinguishable from the other treatment groups when the first samples were taken at day 21. However, when the noise treatment ceased at day 18, their rate of telomere loss may have decreased, so that the rate of loss from that point matched that of the control birds. Alternatively, it may be that noise-exposed parents invest more in parental care, which may buffer noise-induced telomere loss, and as a result telomere length in their offspring is not significantly different to offspring from the control group. Such an adjustment of parental care could, at least partly, be triggered by the chicks, e.g., hatchlings increase the amplitude of their begging calls in noise [48], which may increase feeding rates by the parents. Additionally, it could be that the parents in noise habituated to the noise before their eggs were produced, or offspring hatched, then the lack of an effect of noise exposure on the young from noise-exposed parents might reflect this habituation, whereas in the JNoise treatment, the noise exposure may have occurred at a more vulnerable time in the young birds’ lives. However, whether the parents were able to habituate to noise is unknown. While they had noise exposure for 3 weeks before nest building began, the noise treatment also began for them at a sensitive time in their life histories. It was present as soon as they were added to a new mixed sex aviary. Therefore, they were in an early stage of pair formation and getting used to a new flock composition, which is also a vulnerable period.

Telomere length in zebra finches is thought to be heritable [49], and this is supported by our data, wherein parents explain 27% of the variation in our model. However, whether rates of telomere attrition are also heritable is not clear. We found that juveniles exposed to noise post-fledging had higher rates of attrition, even though their telomere length at day 21 was not different from the other groups (Additional file 1: Figure S1). This suggests that environmental conditions, in this case traffic noise, have an impact on the attrition rate of telomeres in juvenile zebra finches, regardless of inherited differences in telomere length. However, the exact mechanisms underlying the differences observed between our groups, and the degree to which heritability may impact rates of telomere loss independent of environmental conditions needs further study.

The first sample in our study was taken when juveniles were 21 days old post-hatch, around 10 days after the sampling day in other studies [30, 31]. In contrast to this prior research [30, 31], our results showed that traffic noise exposure did not have an effect on telomere length. That our birds did not respond to traffic noise exposure in the same way as the birds in these previous studies may be an indicator of species differences in sensitivity to traffic noise, or may represent a difference between how captive birds and wild birds respond to noise. It may also be that in our study, parents were exposed to noise continuously (like in the city) and while our playback consisted of randomized, unpredictable fluctuations in noise, parents may have habituated more to this chronic noise condition than to shorter daily periods of playback, such as in Meillère et al. (2015).

## Conclusions

Our study contributes critical new data to our understanding of the long-term effects of traffic noise pollution on avian health and fitness. We show that chronic exposure to realistic levels of traffic noise increases rates of telomere loss in older, but not very young juvenile zebra finches. Since telomere length has been shown to predict longevity in zebra finches [17], a noise-induced increase of telomere attrition rate may serve as a biomarker for reduced long-term survival, which, eventually, may even affect population dynamics of birds in noise polluted areas. Previous evidence in juvenile great tits suggests that urban environments increase rates of telomere loss [32]. However, our study suggests that anthropogenic noise alone, independent from the many other urban factors, increases telomere loss and may contribute to organismal ageing. Urbanization consists of a complex suite of ecological changes, and our study is a first step towards identifying the causal mechanisms that may underlie differences observed between urban dwellers and their rural conspecifics. As suggested by our findings, it is essential to consider developmental stage and parental effects when studying these mechanisms and how they ultimately affect eco-evolutionary processes.

## Additional file

Additional file 1: **Table S1.** Overview of aviaries, rooms in which the aviaries were placed, dates of experiments, and treatments. PNoise (the parents were exposed to noise during breeding, egg-laying and nestling care periods, which also meant that nestlings were exposed to noise until they left the nest, ~18 days post-hatch), JNoise (juvenile birds were exposed to noise exposure from fledging throughout the sensory motor learning period, 18-120 days post-hatch) and control (parents and juveniles not exposed to noise at any time point). **Table S2.** Statistical models and their respective Akaike Information Criterion (AIC). **Table S3.** Outcome of linear models testing the effects of noise on the telomere length of juvenile zebra finches that had parents exposed to noise (PNoise), or that were themselves exposed to noise (JNoise) and a no-noise control group. The asterisks represent “significant” differences in the frequentist’s sense, i.e. when the 95% credible intervals did not overlap zero [39]. The telomere length values were calculated according to Pfaffl, 2001. **Figure S1. **Scatter plot of telomere length values of zebra finches at day 120 against day 21. The colours represent the treatments: parents exposed to noise (orange circles), juveniles exposed to noise themselves (red circles) and control (blue circles). The lines show the regression lines per treatment. **Figure S2.** scatter plot of telomere length values of zebra finches obtained with Cawthon method against Pfaffl method. According to Pearson correlation test the values are highly correlated (0.95, *p*-value < 2.2e-16). **Table S4.** Outcome of linear models testing the effects of noise on the telomere length of juvenile zebra finches that had parents exposed to noise (PNoise), or that were themselves exposed to noise (JNoise) and a no-noise control group. The asterisks represent “significant” differences in the frequentist’s sense, i.e. when the 95% credible intervals did not overlap zero [39]. This data did not include the outline point from treatment JNoise. (PDF 137 kb)
