# Supplementary material for: Timing matters: traffic noise accelerates telomere loss rate differently across developmental stages
Source: Front Zool. 2018 Aug 28;15:29. doi: 10.1186/s12983-018-0275-8 (PMC6112141; doi:10.1186/s12983-018-0275-8)
Supplement: Supplementary file 1 — Table S1. Overview of aviaries, rooms in which the aviaries were placed, dates of experiments, and treatments. PNoise (the parents were exposed to noise during breeding, egg-laying and nestling care periods, which also meant that nestlings were exposed to noise until they left the nest, ~18 days post-hatch), JNoise (juvenile birds were exposed to noise exposure from fledging throughout the sensory motor learning period, 18-120 days post-hatch) and control (parents and juveniles not exposed to noise at any time point). Table S2. Statistical models and their respective Akaike Information Criterion (AIC). Table S3. Outcome of linear models testing the effects of noise on the telomere length of juvenile zebra finches that had parents exposed to noise (PNoise), or that were themselves exposed to noise (JNoise) and a no-noise control group. The asterisks represent “significant” differences in the frequentist’s sense, i.e. when the 95% credible intervals did not overlap zero [39]. The telomere length values were calculated according to Pfaffl, 2001. Figure S1. Scatter plot of telomere length values of zebra finches at day 120 against day 21. The colours represent the treatments: parents exposed to noise (orange circles), juveniles exposed to noise themselves (red circles) and control (blue circles). The lines show the regression lines per treatment. Figure S2. scatter plot of telomere length values of zebra finches obtained with Cawthon method against Pfaffl method. According to Pearson correlation test the values are highly correlated (0.95, p-value < 2.2e-16). Table S4. Outcome of linear models testing the effects of noise on the telomere length of juvenile zebra finches that had parents exposed to noise (PNoise), or that were themselves exposed to noise (JNoise) and a no-noise control group. The asterisks represent “significant” differences in the frequentist’s sense, i.e. when the 95% credible intervals did not overlap zero [39]. This data did not include the outline p [file 12983_2018_275_MOESM1_ESM.pdf]

Timing matters: traffic noise accelerates telomere loss rate differently across developmental stages

A. M. Dorado-Correa, S.A. Zollinger, B. Heidinger and H. Brumm

Frontiers in Zoology 2018 **15**:29

Supporting information

Table S1. Overview of aviaries, rooms in which the aviaries were placed, dates of experiments, and treatments. PNoise (the parents were exposed to noise during breeding, egg-laying and nestling care periods, which also meant that nestlings were exposed to noise until they left the nest, ~18 days post-hatch), JNoise (juvenile birds were exposed to noise exposure from fledging throughout the sensory motor learning period, 18-120 days post-hatch) and control (parents and juveniles not exposed to noise at any time point).

Group (aviary)	Room	Dates	Treatment
1	1	Apr 2014-Sept 2014	PNoise
2	1	Apr 2014-Sept 2014	PNoise
3	1	Apr 2014-Sept 2014	PNoise
4	2	Apr 2014-Sept 2014	Control
5	2	Apr 2014-Sept 2014	Control
6	2	Apr 2014-Sept 2014	Control
1	1	Nov 2014-Mar 2015	Control
2	1	Nov 2014-Mar 2015	Control
3	1	Nov 2014-Mar 2015	Control
4	2	Nov 2014-Mar 2015	PNoise
5	2	Nov 2014-Mar 2015	PNoise
6	2	Nov 2014-Mar 2015	PNoise
7	3	Mar 2015- Aug 2015	JNoise
8	3	Mar 2015- Aug 2015	JNoise
9	3	Mar 2015- Aug 2015	JNoise

Table S2. Statistical models and their respective Akaike Information Criterion (AIC).

Model	AIC
Interaction between all fixed effects	373.86
Non interaction between all fixed variables	339.30
Interaction between treatment, sex and age	337.89
Interaction between treatment and age	316.16

Table S3. Outcome of linear models testing the effects of noise on the telomere length of juvenile zebra finches that had parents exposed to noise (PNoise), or that were themselves exposed to noise (JNoise) and a no-noise control group. The asterisks represent "significant" differences in the frequentist's sense, i.e. when the 95% credible intervals did not overlap zero [39]. The telomere length values were calculated according to Pfaffl, M.W. (2001) A new mathematical model for relative quantification in real-time RT–PCR. Nucleic Acids Res 29:e45.

**Parameters**	**Estimate (β)**	**95% CI**
**Fixed effects**		
(Intercept)	0.10	-0.17,0.35
Parents in noise (PNoise)	0.06	-0.01,0.12
Offspring in noise (JNoise)	0.08	-0.03,0.19
Sex	0.03	-0.02,0.07
Age	-0.10	-0.17,-0.03*
Breeding round	0.03	-0.03,0.08
Mass	0.008	-0.01,0.03
PNoise x age	-0.08	-0.17,0.01
JNoise x age	-0.38	-0.63,-0.11*
**Random effects**	**Std. Dev (σ^2)**	
Individual ID (Intercept)	0.00	
Mother ID (Intercept)	0.08	
Father ID (Intercept)	0.09	
Group (Intercept)	0.01	
Plate	0.20	

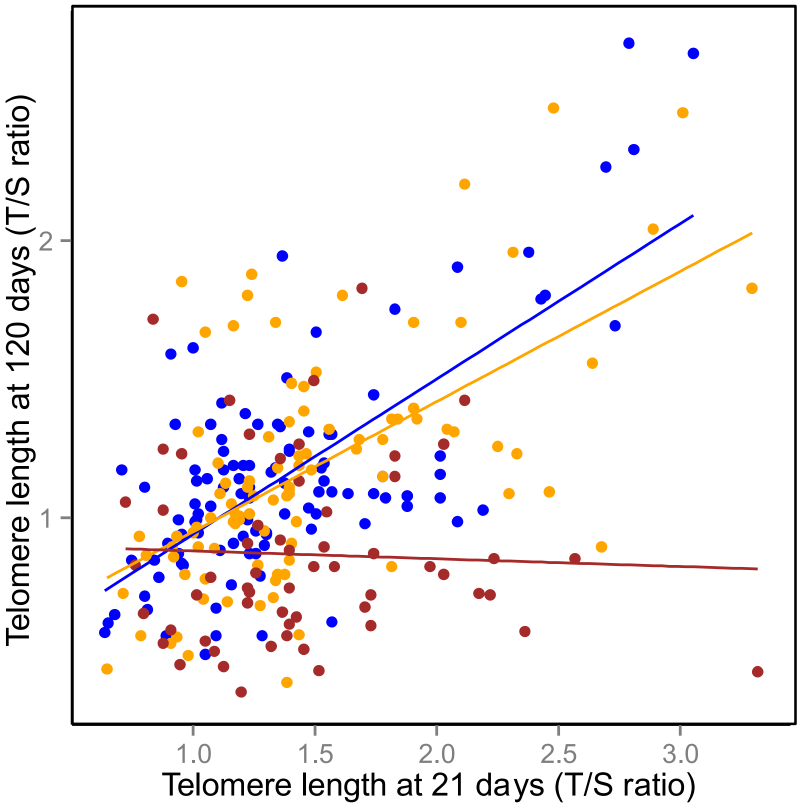

Figure S1. Scatter plot of telomere length values of zebra finches at day 120 against day 21. The colours represent the treatments: parents exposed to noise (orange circles), juveniles exposed to noise themselves (red circles) and control (blue circles). The lines show the regression lines per treatment.

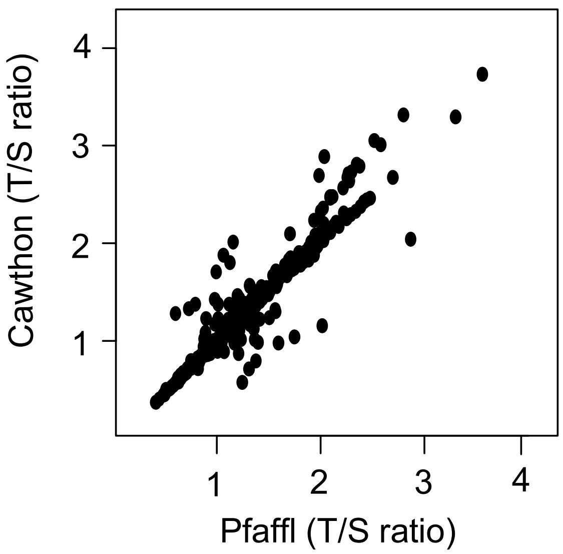

Figure S2. Telomere length values of zebra finches obtained with Cawthon´s method [34] against Pfaffl´s method (Pfaffl 2001 Nucleic Acids Res 29:e45). According to a Pearson correlation test the values are highly correlated (r=0.95, p-value < 0.0001).

Table S4. Outcome of linear models testing the effects of noise on the telomere length of juvenile zebra finches that had parents exposed to noise (PNoise), or that were themselves exposed to noise (JNoise) and a no-noise control group. The asterisks represent “significant” differences in the frequentist’s sense, i.e. when the 95% credible intervals did not overlap zero [39]. This data does not include the outlying point from treatment JNoise.

**Parameters**	**Estimate (β)**	**95% CI**
**Fixed effects**		
(Intercept)	0.12	-0.15,0.38
Parents in noise (PNoise)	0.06	-0.01,0.12
Offspring in noise (JNoise)	0.03	-0.07,0.15
Sex	0.03	-0.01,0.07
Age	-0.20	-0.28,-0.12*
Breeding round	0.03	-0.03,0.08
Mass	0.01	-0.007,0.03
PNoise x age	-0.06	-0.15,0.02
JNoise x age	-0.32	-0.63,-0.02*
**Random effects**	**Std. Dev (σ^2)**	
Individual ID (Intercept)	0.04	
Mother ID (Intercept)	0.08	
Father ID (Intercept)	0.09	
Group (Intercept)	0	
Plate	0.22	
Residual	0.22	
